# Distribution of Hemoglobin Variants: A Retrospective Study Based on Hemoglobin Electrophoresis Results From a University Hospital in Madagascar

**DOI:** 10.7759/cureus.111094

**Published:** 2026-06-18

**Authors:** Jocia Fenomanana, Faralahy H Rakotonjafiniarivo, Fanirisoa Rasolozakandrainibe, Stephania Niry Manantsoa, Anjarasoa Diamondra Malalanirina, Miora Koloina Ranaivosoa, Aimée Olivat Rakoto Alson

**Affiliations:** 1 Medical Biology, Centre Hospitalier Universitaire Andrainjato, Fianarantsoa, MDG; 2 Faculty of Medicine, University of Fianarantsoa, Fianarantsoa, MDG; 3 Medical Biochemistry, Centre Hospitalier Universitaire Joseph Ravoahangy Andrianavalona, Antananarivo, MDG; 4 Faculty of Medicine, University of Antananarivo, Antananarivo, MDG; 5 Public Health, Centre Hospitalier Universitaire Andrainjato, Fianarantsoa, MDG; 6 Medical Biology/Hematology, University of Antananarivo, Antananarivo, MDG; 7 Medicine, Centre Hospitalier Universitaire Andrainjato, Fianarantsoa, MDG; 8 Medical Biochemistry, University of Antananarivo, Antananarivo, MDG; 9 Hematology, University of Antananarivo, Antananarivo, MDG

**Keywords:** hemoglobin electrophoresis, hemoglobinopathies, madagascar, sickle cell disease, thalassemia

## Abstract

Introduction: Inherited hemoglobin disorders, including sickle cell disease and thalassemias, are a growing public health burden, particularly in low-resource countries. Data from Madagascar remain limited. This study describes the distribution of hemoglobin variants and evaluates methodological considerations using agarose gel electrophoresis in patients referred for laboratory analysis.

Methods: A retrospective study was conducted on 553 patients (2014-2019) referred for hemoglobin analysis. Hemoglobin variants were identified using agarose gel electrophoresis (Hydrasys system, Sebia). Demographic data and temporal trends were analyzed.

Results: Normal hemoglobin profiles were observed in 133 patients (24.1%). HbAS was the most frequent variant (247; 44.7%), followed by HbSS (105; 19.0%). Isolated alpha-thalassemia and beta-thalassemia were rare, 1 (0.2%) and 7 (1.3%). Combined hemoglobinopathies were observed, predominantly HbAS with alpha-thalassemia (29; 5.2%) and HbAS with beta-thalassemia (17; 3.1%). Agarose gel electrophoresis provided accessible detection of common variants but may underestimate or misclassify rare or complex variants due to co-migration and limited quantification.

Conclusion: Sickle cell-related hemoglobinopathies are highly prevalent in Madagascar, with significant genetic heterogeneity including combined forms. While agarose gel electrophoresis is practical in resource-limited settings, complementary techniques such as high-performance liquid chromatography or molecular analysis are recommended for accurate diagnosis. These findings support the need for strengthened screening programs and improved diagnostic capacity to better manage hemoglobinopathies in Madagascar.

## Introduction

Inherited hemoglobin disorders constitute a major emerging global public health burden, particularly in low-resource countries [1]. Hemoglobinopathies, including sickle cell disease and thalassemia syndromes, represent a major global health burden, particularly in sub-Saharan Africa, where they contribute significantly to childhood morbidity and mortality [2].

In Madagascar, the epidemiology of hemoglobinopathies remains insufficiently characterized despite growing evidence of their clinical and public health importance. Previous studies have reported variable prevalence rates depending on geographic regions, with higher frequencies observed in coastal areas [3]. For instance, Maeder et al. reported a prevalence of sickle cell trait of 23.8% and a prevalence of sickle cell disease of 2.4% among 807 febrile children living in a rural area of southeastern Madagascar. Similarly, the presence of sickle cell trait among blood donors was also identified, highlighting the circulation of hemoglobin variants in the general population [4]. However, these data remain limited and heterogeneous, underscoring the need for larger laboratory-based studies.

Accurate identification of hemoglobin variants is essential for diagnosis, patient management, and genetic counseling. While high-performance liquid chromatography (HPLC) is considered a reference method, agarose gel electrophoresis remains widely used in resource-limited settings due to its accessibility and reliability for detecting common variants such as HbA and HbS [5].

The present study primarily aimed to determine the distribution of hemoglobin variants among patients referred for hemoglobin electrophoresis over a six-year period (2014-2019) in Madagascar using agarose gel electrophoresis. Secondary objectives were to evaluate demographic characteristics, assess temporal trends in variant distribution, and discuss the utility and limitations of agarose gel electrophoresis for the diagnosis of hemoglobinopathies in a resource-limited setting.

## Materials and methods

Study design and population

This was a retrospective descriptive study conducted on patients referred for hemoglobin analysis at the biochemistry laboratory of University Hospital Joseph Ravoahangy Andrianavalona in Antananarivo, Madagascar, from 2014 to 2019. Demographic and laboratory data were collected from laboratory records. Inclusion criteria were all patients who underwent hemoglobin electrophoresis during the study period and for whom demographic information and hemoglobin analysis results were available. Exclusion criteria included records with incomplete data, defined as missing key demographic variables and/or hemoglobin analysis results, duplicate records, and samples considered unsuitable for interpretation. Patients with incomplete data were excluded from the analysis.

Diagnostic criteria

Hemoglobin variants were identified based on the migration profiles obtained by agarose gel electrophoresis according to the manufacturer’s instructions and routine laboratory interpretation procedures. Variant classification was based on the electrophoretic mobility and relative distribution of hemoglobin fractions. In cases showing atypical or complex electrophoretic patterns suggestive of combined hemoglobinopathies, interpretation was limited to the electrophoretic findings, as complementary investigations such as molecular analysis, iron studies, or complete blood count were not systematically available. Therefore, definitive differentiation of complex conditions, including sickle cell disease associated with alpha- or beta-thalassemia, could not be established.

Ethical considerations

Anonymized laboratory data were used. Due to the retrospective design of the study and the use of pre-existing anonymized data extracted from the laboratory database without direct patient contact or additional procedures, formal review by a local ethics committee was not required according to institutional requirements. This study was conducted in accordance with the ethical principles of the Declaration of Helsinki, ensuring data confidentiality and respect for human rights. Patients were assigned unique identification numbers to ensure anonymity and protect the collected information. Medical confidentiality was maintained through the use of coded data collection forms.

Hemoglobin analysis

Hemoglobin variants were analyzed by agarose gel electrophoresis using the Hydrasys system (Sebia, Lisses, France), following the manufacturer’s recommendations. The Hydragel 7 Hemoglobin kit (Sebia) was used for separation.

Hemolysates were prepared from whole blood (peripheral venous blood samples collected in EDTA tubes) and applied to agarose gels. Electrophoresis was performed under alkaline conditions, enabling separation of hemoglobin fractions according to their electrophoretic mobility. After migration, gels were stained and scanned using the automated system. Identification of hemoglobin fractions was based on comparison with control materials and reference migration patterns provided by the manufacturer. Internal quality control procedures were applied in accordance with routine laboratory practice. To ensure the reliability of laboratory results, internal quality control samples were analyzed concomitantly with patient samples, and regular preventive maintenance and calibration of the equipment were performed in collaboration with the instrument supplier.

Statistical analysis

The collected data were entered using Microsoft Excel software (Microsoft Corporation, Redmond, USA). After data entry, quality control procedures were performed to identify data entry errors, duplicates, and missing data. Statistical analysis was subsequently carried out using R software version 4.5.1 (R Foundation for Statistical Computing, Vienna, Austria).

A descriptive analysis of the study variables was conducted. Categorical variables were expressed as frequencies and percentages, whereas continuous variables were summarized using means ± standard deviations, or medians with ranges, as appropriate. Temporal trends in hemoglobin variant distribution over the study period were described using descriptive statistics. The results were presented in tables and graphical representations.

## Results

A total of 553 patients were included in the study. The mean age was 19.3 years (range: 0.25-81.92).

Distribution of hemoglobin variants

A normal hemoglobin profile was identified in 24.05% (133) of patients. Among abnormal findings, HbAS was the most frequent variant, accounting for 44.67% (247) of patients, followed by HbSS in 18.99% (105) of patients. Isolated thalassemia was infrequent, with alpha-thalassemia and beta-thalassemia each observed in 1.26% (7) and 1.26% (7) of cases, respectively. Combined hemoglobinopathies were observed in a subset of patients. The most common association was HbAS with alpha-thalassemia 5.24% (29), followed by HbAS with beta-thalassemia (3.07%; 17). Co-inheritance involving HbSS was rare, with HbSS/alpha-thalassemia and HbSS/beta-thalassemia each accounting for 0.72% (4) of patients (Table 1).

**Table 1:** Distribution of hemoglobin variants (n = 553)

Variants	Number of Cases	Percentage (%)
Normal	133	24.05
Heterozygous sickle cell (HbAS)	247	44.67
Homozygous sickle cell (HbSS)	105	18.99
Alpha-thalassemia	7	1.26
Beta-thalassemia	7	1.26
HbAS + Alpha-thalassemia	29	5.24
HbAS + Beta-thalassemia	17	3.07
HbSS + Alpha-thalassemia	4	0.72
HbSS + Beta-thalassemia	4	0.72
Total	553	100

Temporal trends

**Figure 1:**
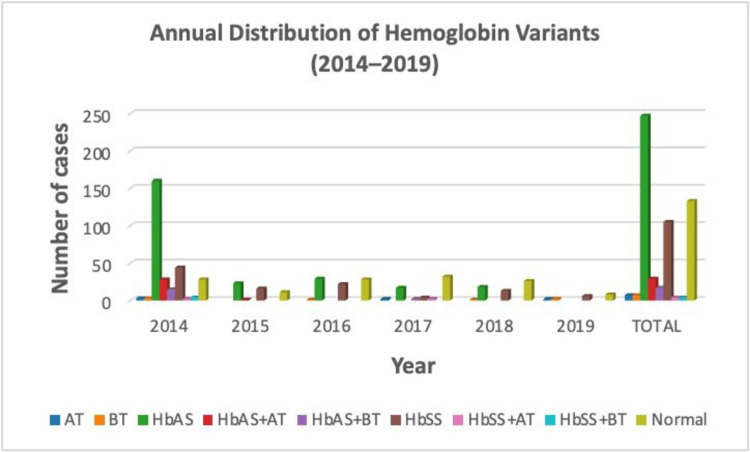
Annual distribution of hemoglobin variants AT: Alpha-thalassemia; BT: beta-thalassemia

Descriptive analysis by year suggested variations in both patient demographics and the distribution of hemoglobin variants over time (Figure 1). 

## Discussion

In this study, we described the distribution of hemoglobin variants in a cohort of 553 patients referred for laboratory investigation over a five-year period. Our findings highlight a high prevalence of sickle cell-related hemoglobinopathies, with HbAS being the most frequent variant (44.67%; 247), followed by HbSS (18.99%; 105 patients).

The predominance of HbAS observed in our cohort is consistent with data reported in sub-Saharan Africa, where the sickle cell trait is highly prevalent due to the selective advantage conferred against malaria [6]. Similar findings have been reported in Madagascar. In a study conducted by Maeder et al., the prevalence of HbAS reached 23.8% among febrile children, although this figure remains lower than that observed in our study [4]. This difference may be explained by the hospital-based nature of our population, which likely includes a higher proportion of individuals referred for suspected hemoglobin disorders.

The proportion of HbSS in our study is also notable and suggests a substantial burden of sickle cell disease in the studied population. This finding is in line with previous reports indicating that sickle cell disease remains underdiagnosed in Madagascar despite its clinical significance. Fenomanana et al. demonstrated the presence of hemoglobin S among blood donors, highlighting the circulation of the gene in the general population, although at lower frequencies due to the selection of healthier individuals [3].

Isolated thalassemia was rare in our cohort, with both alpha- and beta-thalassemia accounting for approximately 1.3% (7) of cases. This low prevalence is consistent with the limited data available in Madagascar but contrasts with regions such as the Mediterranean, Middle East, and Southeast Asia, where thalassemia syndromes are highly prevalent [7]. However, the presence of combined forms, particularly HbAS associated with alpha- or beta-thalassemia, underscores the genetic heterogeneity of hemoglobinopathies in this setting.

The frequency of combined hemoglobinopathies observed in our study, notably HbAS/alpha-thalassemia and HbAS/beta-thalassemia, is clinically relevant, as these interactions may modulate disease severity and complicate diagnosis. Similar associations have been reported in African populations, where co-inheritance patterns contribute to phenotypic [8-10].

Temporal analysis suggested variability in both patient demographics and hemoglobin variant distribution over the study period. Although no formal trend analysis was performed, these fluctuations may reflect changes in referral patterns, increased awareness, or improved access to diagnostic testing over time.

From a methodological perspective, agarose gel electrophoresis proved to be a useful and accessible tool for the detection of common hemoglobin variants in a resource-limited setting [10,11]. However, as previously reported, this technique has limitations, particularly regarding the resolution of variants with similar electrophoretic mobility and the accurate quantification of hemoglobin fractions [12,13]. The absence of complementary methods such as HPLC or molecular analysis may have led to underestimation or misclassification of certain variants [14,15].

Limitations of the study

This study has some limitations. Its retrospective design may be associated with incomplete data and selection bias, as only patients referred for testing were included; therefore, the findings may not be representative of the general population and should not be extrapolated to estimate the national burden of hemoglobinopathies in Madagascar. In addition, demographic and complementary laboratory data, including complete blood count and iron studies, were not systematically available, limiting a more comprehensive characterization of patient profiles. The absence of confirmatory techniques and the reliance on agarose gel electrophoresis alone reduced the precision of variant identification and limited the reliable differentiation of complex hemoglobinopathy profiles, particularly cases involving possible co-inheritance of sickle cell disease with alpha- or beta-thalassemia. These methodological constraints may also have affected the interpretation of temporal patterns and the estimation of thalassemia frequency. Furthermore, statistical analyses were mainly descriptive and did not allow assessment of associations or causal relationships.

Despite these limitations, this study provides valuable laboratory-based data on the distribution of hemoglobin variants among patients referred for testing in Madagascar. It highlights the importance of strengthening diagnostic capacities and implementing broader population-based epidemiological studies to better characterize the burden of hemoglobinopathies in the country.

## Conclusions

This study provides an overview of the distribution of hemoglobin variants in a Malagasy cohort referred for laboratory investigation. A high prevalence of sickle cell-related hemoglobinopathies was observed, with HbAS as the predominant variant, followed by a substantial proportion of HbSS cases. Although isolated thalassemia was uncommon, the presence of combined hemoglobinopathies highlights the genetic diversity of these disorders in this setting. These findings should be interpreted in the context of a laboratory-referred population and may not be representative of the general Malagasy population.

Despite methodological limitations, these findings contribute to the limited body of data on hemoglobinopathies in Madagascar and underscore the need for broader epidemiological studies to better define their national burden.
